# Correlation between hematological parameters and outcome in patients with locally advanced cervical cancer treated by concomitant chemoradiotherapy

**DOI:** 10.1002/cam4.3465

**Published:** 2020-09-20

**Authors:** Christine Gennigens, Marjolein De Cuypere, Laurence Seidel, Johanne Hermesse, Annelore Barbeaux, Frédéric Forget, Adelin Albert, Guy Jerusalem, Frédéric Kridelka

**Affiliations:** ^1^ Department of Medical Oncology CHU Liège Liège Belgium; ^2^ Department of Obstetrics and Gynaecology CHU Liège Belgium; ^3^ Department of Biostatistics CHU Liège and Liège University Liège Belgium; ^4^ Department of Radiotherapy CHU Liège Liège Belgium; ^5^ Department of Medical Oncology CHR East Belgium Verviers Belgium; ^6^ Department of Medical Oncology Libramont Hospital Libramont Belgium; ^7^ Department of Medical Oncology CHU Liège and Liège University Liège Belgium; ^8^ Department of Obstetrics and Gynaecology CHU Liège and Liège University Liège Belgium

**Keywords:** chemoradiotherapy, hemoglobin, locally advanced cervical cancer, neutrophil, transfusion, white blood cell

## Abstract

**Background:**

Hemoglobin (Hb), white blood cell (WBC), and polymorphonuclear neutrophil (PMN) blood counts may be correlated with outcomes in patients with locally advanced cervical cancer.

**Methods:**

Hb, WBC, and PMN counts were measured at diagnosis and during concomitant cisplatin‐based chemoradiotherapy (CCRT) in a retrospective sample of 103 patients between 2010 and 2017. Red blood cell (RBC) transfusions were also recorded. The associations between hematological variables and patient overall survival (OS) and recurrence‐free survival (RFS) were assessed by Cox regression models.

**Results:**

The 3‐year OS and RFS rates were 81.4% and 76.8%, respectively. In addition to tumor size and smoking, OS and RFS were found to be significantly associated with changes in WBC and PMN counts from the first to the last cisplatin cycle. Hb count throughout the treatment and RBC transfusions were not predictive of outcome.

**Conclusions:**

This study found no association between Hb count or RBC transfusions and outcome. The daily practice of maintaining the Hb count above 12 g/dL during CCRT should be weighed against the potential risks of transfusions. Drops in WBC and PMN counts during treatment positively impacted OS and RFS and could, therefore, serve as biomarkers during CCRT to adapt the follow‐up and consider the need for adjuvant systemic treatments.

## Graphical abstract

In patients with locally advanced cervical cancer. Daily practice of maintaining the Hb count above 12 g/dL during CCRT should be weighed against potential risks of transfusions. Drops in WBC and PMN counts during treatment positively impacted OS and RFS

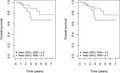

## 1 BACKGROUND

Cervical cancer (CC) is the fourth most common cancer in women after breast, colorectal, and lung cancers.^1^ Concomitant cisplatin‐based chemoradiotherapy (CCRT) followed by image‐guided adaptive brachytherapy (IGABT) is the recommended treatment for patients suffering from locally advanced cervical cancer (LACC) (stage ≥ IB2 according to the International Federation of Gynaecology and Obstetrics, FIGO 2009). The treatment shifted from radiotherapy (RT) alone to CCRT following the publication of five randomized controlled trials that demonstrated an improvement in overall survival (OS) and recurrence‐free survival (RFS) with the addition of radiosensitizing concomitant chemotherapy (CT).^2^, ^3^ For patients with metastatic lymph nodes (LNs) in the para‐aortic (PAo) region, the RT field is extended to the PAo region with the aim of adequately covering this nodal area. Lymphatic dissemination can be explored by positron emission tomography/computed tomography (PET/CT) using ﻿^18^F‐2’‐deoxy‐2’‐fluorodeoxyglucose ([^18^F]FDG) and by PAo laparoscopic lymphadenectomy.^4^ Several patient‐, tumor ‐ or treatment‐related variables have been found to be predictive of outcome after RT alone or CCRT, such as age, HPV status, tumor size, histology, FIGO stage, LN involvement, and initial [^18^F]FDG‐PET/CT avidity.^5^, ^6^, ^7^ Associations between the hemoglobin (Hb) count recorded during CCRT and patient outcome, such as OS and RFS, have also been reported in various retrospective trials but with conflicting results.^8^, ^9^, ^10^, ^11^ Although the only prospective trial evaluating the benefit of correcting the Hb count via red blood cell (RBC) transfusions in CC was clearly underpowered for this purpose, the study findings suggested that increasing the Hb count to >12 g/dL could decrease the risk of local relapse.^12^ After this publication, the approach was generally accepted and used in daily practice. In contrast, other trials (colorectal, ovarian, bladder, and cervical cancers) suggested that perioperative RBC transfusions could increase the risk of recurrence.^13^, ^14^, ^15^, ^16^, ^17^ Other additional risks of RBC transfusions, such as infection, allergic reaction, higher thrombotic risk, and immunosuppression, should also be considered.^18^

Far less is known about the clinical impact of white blood cell (WBC) or polymorphonuclear neutrophil (PMN) counts on outcome in the treatment of patients with LACC. Recent articles demonstrated that neutrophils promote tumor resistance to RT and that they can play an active role in tumor growth and distant tumor dissemination.^19^ They are also involved in the pro‐inflammatory tumor microenvironment (TME) induced by immune‐stimulating effects of RT.^20^ Other observations underlined that chemotherapy‐induced neutropenia (CIN) appears to be more than just a hematoxicity predictive of infectious complications. It could also be a marker of response and/or survival in patients treated with CT for several types of cancers.^21^, ^22^

This study aimed to investigate the impact of Hb, WBC, and PMN counts throughout treatment in a cohort of patients with LACC who received cisplatin‐based CCRT followed by IGABT.

## 2 MATERIALS AND METHODS

### 2.1 Study design

This was a retrospective, non‐controlled, cohort study based on patients with LACC (stage ≥ IB2 according to FIGO 2009) who received CCRT between January 2010 and May 2017 followed by IGABT. [^18^F]FDG‐PET/CT, PAo laparoscopic lymphadenectomy, and brachytherapy were all performed at the University Hospital of Liège, Liège, Belgium. Based on a medical chart review, 134 patients were identified to be enrolled in the study, but 31 were excluded for various reasons: rare histology (n = 15), non‐cisplatin‐containing CT regimen (n = 8), and unknown date of CCRT initiation (n = 8). The remaining 103 patients constituted the study population. The period during which the patients were treated necessitated the choice of the FIGO 2009 staging system rather than the revised 2018 FIGO classification. Only patients with histology of squamous cell carcinoma (SCC) or adenocarcinoma (AC) were included in the study.

### 2.2 Chemotherapy

All patients were treated with cisplatin‐based CT. They received at least one cycle of weekly intravenous concomitant cisplatin (40 mg/m^2^) with a target of five or six cycles during external beam radiation (EBRT). Laboratory tests were performed weekly during the course of CCRT.

### 2.3 Radiotherapy

Patients were treated with three‐dimensional conformal RT or by intensity‐modulated RT or volumetric‐modulated arc therapy techniques. Fractions of 1.8 to 2 Gy were delivered up to five times a week. Extended‐field RT to treat the PAo region was based on [^18^F]FDG‐PET/CT and laparoscopic LN staging. Radiation boosts up to 60 Gy to pathologic nodes was also delivered. Brachytherapy (BT) was administered with a pulse‐dose rate technique with intracavitary or intracavitary and interstitial techniques. All patients underwent CT‐scan and magnetic resonance imaging (MRI) simulations for EBRT and BT planning, respectively. Total RT time was defined as the first day of EBRT to the last day of BT. From April 2013, all patients were treated by intensity‐modulated RT or volumetric‐modulated arc therapy. Whenever indicated, interstitial BT was applied.

### 2.4 Follow‐up and outcomes

Patients were followed up every 3 months for the first 2 years, every 6 months for years 3 to 5, and annually thereafter. At 3 months posttreatment, [^18^F]FDG‐PET/CT and MRI were performed to check the treatment response. Assessments during the follow‐up period included general and pelvic examinations, biology and, in case of suspicion of relapse, a biopsy of accessible tumor sites and/or imaging studies at each time point. A [^18^F]FDG PET/CT was also performed once a year for 3 years in cases of positive LNs at the time of diagnosis.

The study outcomes consisted primarily of OS and RFS. Cancer‐specific survival (CSS), pelvic failure‐free survival (PFFS), and freedom from distant metastasis (FFDM) were also considered. All intervals were calculated from the start of CCRT until treatment failure or the last date of follow‐up. OS was defined as the time to death from any cause. RFS was defined as the time to first imagery and/or pathologic evidence of disease recurrence locally, within the lymph nodes (pelvic, PAo or distant) or at distant sites. PFFS was defined as the time without local and/or LN pelvic recurrence. FFDM was defined as the time without distant LNs and/or visceral recurrence.

### 2.5 Statistical analysis

The results are expressed as the median and range for quantitative variables and as the frequency table (number, percent) for categorical variables. A log‐transform was applied to most hematological parameters to normalize the distribution. Groups were compared by classical one‐way analysis of variance or the non‐parametric Kruskal‐Wallis test. For comparing proportions, the chi‐square or Fisher exact test was used. Linear regression was applied to assess the relationship between two quantitative variables. Cox regression analysis was used to test the relationship between survival outcomes and potential patient‐, tumor‐, and treatment‐related factors. The association was measured by the hazard ratio (HR) and its 95% confidence interval (95% CI). Among the predictive factors of the outcome, special attention was paid to hematological blood count variables at diagnosis and during CT cycles. In particular, the change in hematological variables during CT was expressed as the difference in counts recorded at the baseline (first) cycle (CB) and last cycle (CL) of chemotherapy on a log‐scale (except for Hb where original values were used). Since log (CB) – log (CL) = log (CB/CL), the results can be interpreted in terms of the ratio of CB/CL by taking the exponential of the difference. All calculations were always performed on the maximum number of data available, and missing values were neither replaced nor imputed. Given the limited number of patients and the number of potential covariates, statistical analyses were mostly univariate. The results were considered significant at the 5% critical level (*P* < .05). Statistical analysis was performed using SAS version 9.4 (SAS Institute, NC, USA) and R version 3.6 (graphics).

## 3 RESULTS

### 3.1 Baseline patient and tumor characteristics

The clinical and pathological characteristics of the 103 study patients are shown in Table 1. The median age at diagnosis was 50 years (range 25‐81 years), and the median BMI was 24.2 kg/m^2^ (range 15.8‐23.7 kg/m^2^). Fifty‐seven patients were smokers (61.3%). The majority of the patients had a performance status (PS) at diagnosis of 0 (75.7%) or 1 (22.3%). The median tumor size measured by MRI at diagnosis was 47 mm (range 15‐106 mm). Most patients had FIGO 2009 stage II (60.2%) compared to stage I (21.4%) or stage III (13.3%) disease. SCC was the most frequent histology (88.3%). Based on [^18^F]FDG‐PET/CT and laparoscopic PAo lymphadenectomy staging, 16.8% of patients had a positive pelvic node status, and 18.8% were positive in the PAo region.

**Table 1:** Clinical and pathological characteristics of patients with LACC included in the study (N = 103)

Characteristic	Category	Number (%)	Median	Range
Age (y)			50	25‐81
BMI (kg/m^2^)			24.2	15.8‐23.7
Smoking	Yes	57 (61.3)		
	No	36 (38.7)		
Performance status	0	78 (75.3)		
	1	23 (22.3)		
	2	2 (1.9)		
Tumour size (mm)			47	15‐106
FIGO stage	IB2	21 (21.4)		
	IIA	14 (14.3)		
	IIB	45 (45.9)		
	IIIA	2 (2.0)		
	IIIB	11 (11.2)		
	IVA	5 (5.1)		
Histology	SCC	91 (88.3)		
	AC	12 (11.7)		
LN pelvic status	Negative	79 (83.2)		
	Positive	16 (16.8)		
LN para‐aortic status	Negative	78 (81.3)		
	Positive	18 (18.8)		

Abbreviations: AC, adenocarcinoma; BMI, body mass index; FIGO, International Federation of Gynecology and Obstetrics; LN, lymph node**;** SCC, squamous cell carcinoma.

### 3.2 Chemotherapy

All patients treated with CCRT received at least one cycle of cisplatin‐based CT with a 40 mg/m^2^/week schedule. The majority of the patients received five (40.4%) or six (39.4%) cycles of cisplatin. The median number of cycles was 5 (range 1‐6).

### 3.3 Radiotherapy

Patients received a median dose of EBRT of 45 Gy (range 40‐50.4 Gy). The majority of the patients received 45 Gy (52%) or 50.4 Gy (46.1%). The median duration of EBRT was 37 days (range 28‐59 days). Thirty‐three patients (32.4%) received a nodal boost of RT with a median dose of 10 Gy. Twenty‐one patients (20.4%) received treatment of the pelvic and of the PAo region. The median cumulative dose of the full treatment by RT was 85 Gy administered over 51 days (range 31‐94 days). The median time between diagnosis based on biopsy and the start of CCRT was 62 days (19‐228 days). Brachytherapy was administered to all patients with a median dose of 35 Gy. Forty‐three (41.7%) and 60 (58.3%) patients received intra‐cavitary and intra‐cavitary and insterstitial BT, respectively.

### 3.4 Follow‐up and survival outcomes

The median follow‐up was 30.1 months (range 4.1‐90.8 months). The OS, RFS, CSS, PFFS and FFDM at 3 years were 81.4%, 76.8%, 84.7%, 92.4% and 76.9%, respectively. During follow‐up, 23 patients (22.3%) developed recurrence. The sites of disease at the time of first relapse were local in 5 patients (4.9%), 1 (1%) in pelvic LN, 6 (5.9%) in para‐aortic LNs, 8 (7.8%) in distant LNs, and visceral in 14 patients (13.7%). In total, 17 patients (16.5%) died, and 3 were alive with recurrent disease (2.9%).

### 3.5 Hemoglobin count

The median Hb count at diagnosis was 13.1 g/dL (range 7.2 −17.4 g/dL). It was higher in smokers than in nonsmokers (13.4 g/dL vs 12.3 g/dl, *P* = .0015) and negatively correlated with tumor size (r = −0.38; *P* = .001). The numbers of patients with Hb counts < 8 g/dL, <9 g/dl, <10 g/dL, <11 g/dL and < 12 g/dL were equal to 1 (1.9%), 4 (3.9%), 5 (4.8%), 13 (12.6%), and 26 (25.2%), respectively. A significant decrease in Hb count was observed between the first and the last cycle of CT (*P* < .0001), and it was 10.8 g/dL (6.7‐14.1 g/dL) during CCRT. In total, 30 patients (31.3%) received RBC transfusions.

### 3.6 WBC and PMN counts at diagnosis and during CT cycles

At diagnosis, the median WBC count (10^3^/mm^3^) was 9.4 (range 2.4‐20). For 35 patients (38%), it was higher than the normal limit of 10.1. For PMN, the median count (10^3^/mm^3^) was 5.7 (range 1.9‐17.5). Forty patients (43.5%) were above the upper normal limit of 6.1. The median counts (10^3^/mm^3^) for lymphocytes and platelets were 1.99 (range 0.4‐4.0) and 298 (range 135‐604), respectively. During CT cycles, WBC and PMN counts decreased from the first to the last cycle of CT (*P* < .0001).

### 3.7 Prediction of survival outcomes

Survival outcomes were analyzed by Cox regression for each study parameter. The results are shown in Tables 2 and 3. Smoking was associated with poorer OS (HR 3.71, 95% CI 1.06‐13.0; *P* = .040), while RFS worsened with larger tumor size (HR, 1.03, 95% CI 1.00‐1.05; *P* = .040). Staging lymphadenectomy tended to be associated with better RFS (*P* = .066). Hb count at diagnosis was neither associated with OS (HR 1.04, 95% CI 0.80‐1.35; *P* = .75) nor with RFS (HR 0.94, 95% CI 0.75‐1.18; *P* = .62), PFFS (*P* = .64) and FFDM (*P* = .78). Similarly, no association was observed with Hb count changes between the first and last CT cycles. For WBC and PMN counts, Cox regression modeling of log‐transformed data showed no associations between their value at diagnosis and any of the survival outcomes (OS, RFS, PFFS, and FFDM). Of note, none of the ratios of WBCs/lymphocytes, PMNs/lymphocytes, and platelets/lymphocytes calculated at diagnosis were correlated with OS and RFS.

**Table 2:** Association of clinical and pathological characteristics of patients with LACC with overall survival and recurrence‐free survival (N = 103)

Covariate	Overall survival			Recurrence‐free survival		
	HR	95% CI	*P*‐value	HR	95% CI	*P*‐value
Age (y)	1.03	0.99‐1.08	.20	0.98	0.95‐1.02	.30
Smoking			.040			.72
Yes vs No	3.71	1.06‐13.0		1.18	0.49‐2.81	
PS	1.37	0.51‐3.66	.53	1.17	0.52‐2.65	.70
Tumour (mm)	1.01	0.99‐1.04	.31	1.03	1.00‐1.05	.037
FIGO stage			.99			.74
II vs IB2	1.12	0.34‐3.68		0.67	0.26‐1.70	
III vs IB2	1.07	0.24‐4.80		0.45	0.09‐1.19	
Histology						
AC vs SCC	0.50	0.07‐3.81	.50	1.34	0.40‐4.51	.64
LN pelvis status						
Positive vs negative	1.00	0.29‐3.52	.99	1.06	0.36‐3.15	.92
LN PAo status						
Positive vs negative	1.04	0.30‐3.68	.95	1.36	0.50‐3.71	.55
Lymphadenectomy						
Yes vs No	0.43	0.14‐1.34	.15	0.42	0.16‐1.06	.066
Duration CCRT + BT (days)	1.02	0.97‐1.06	.45	0.98	0.93‐1.03	.41
Dose all RT (Gy)	1.07	0.99‐1.15	.076	1.01	0.97‐1.05	.62
No. CT cycles	1.30	0.72‐2.38	.39	0.95	0.65‐1.40	.80

Abbreviations: AC, adenocarcinoma; BT, brachytherapy; CCRT, Cisplatin‐based chemoradiotherapy; CI, confidence interval; CT, chemotherapy; FIGO, International Federation of Gynecology and Obstetrics; HR, hazard ratio; LN, lymph node; No, number; PAo, Para‐aortic; PS, performance status; RT, radiotherapy; SCC, squamous cell carcinoma

**Table 3:** Association of hematological parameters of patients with LACC measured at diagnosis and during CT cycles with overall survival and recurrence‐free survival (N = 103)

Covariate	Overall survival			Recurrence‐free survival		
	HR	95% CI	*P*‐value	HR	95% CI	*P*‐value
At diagnosis^a^						
Hb (g/dL)	1.04	0.80‐135	.75	0.94	0.75‐1.18	.62
WBCs (10^3^/mm^3^)	0.57	0.14‐2.34	.44	0.67	0.21‐2.17	.51
PMNs (10^3^/mm^3^)	0.32	0.28‐2.42	.72	0.78	0.31‐1.98	.60
Lymphocytes (10^3^/mm^3^)	0.42	0.12‐1.52	.19	0.52	0.16‐1.72	.29
Platelets (10^3^/mm^3^)	1.02	0.23‐4.53	.98	1.40	0.35‐5.56	.63
WBCs/Lymphocytes	1.27	0.45‐3.61	.66	1.11	0.42‐2.94	.83
PMNs/Lymphocytes	1.23	0.54‐2.76	.62	1.08	0.51‐2.27	.84
Platelets/Lymphocytes	2.15	0.70‐6.62	.18	1.77	0.63‐5.03	.28
RBC transfusion (Yes)	1.15	0.39‐3.33	.80	0.91	0.35‐2.35	.85
Change in CT cycles*						
Hb (g/dL)	1.03	0.75‐1.41	.87	0.83	0.63‐1.09	.17
WBCs (10^3^/mm^3^)	0.29	0.11‐0.76	.011	0.35	0.17‐0.74	.0056
PMNs (10^3^/mm^3^)	0.41	0.19‐0.89	.024	0.44	0.24‐0.82	.0093
Lymphocytes (10^3^/mm^3^)	0.35	0.15‐0.85	.020	0.71	0.33‐1.53	.38
Platelets (10^3^/mm^3^)	0.34	0.11‐1.09	.069	0.43	0.16‐1.12	.083
WBCs/Lymphocytes	0.99	0.41‐2.36	.98	0.59	0.29‐1.18	.14
PMNs/Lymphocytes	0.93	0.47‐1.81	.82	0.63	0.37‐1.09	.098
Platelets/Lymphocytes	1.16	0.51‐2.62	.73	0.81	0.37‐1.76	.59

Abbreviations: CI, confidence interval; Hb, haemoglobin; HR, hazard ratio; PMNs, Polymorphonuclear neutrophils; RBC, red blood cell; WBCs, white blood cells.

^a^ All covariates but Hb were log‐transformed

**Figure 1:**
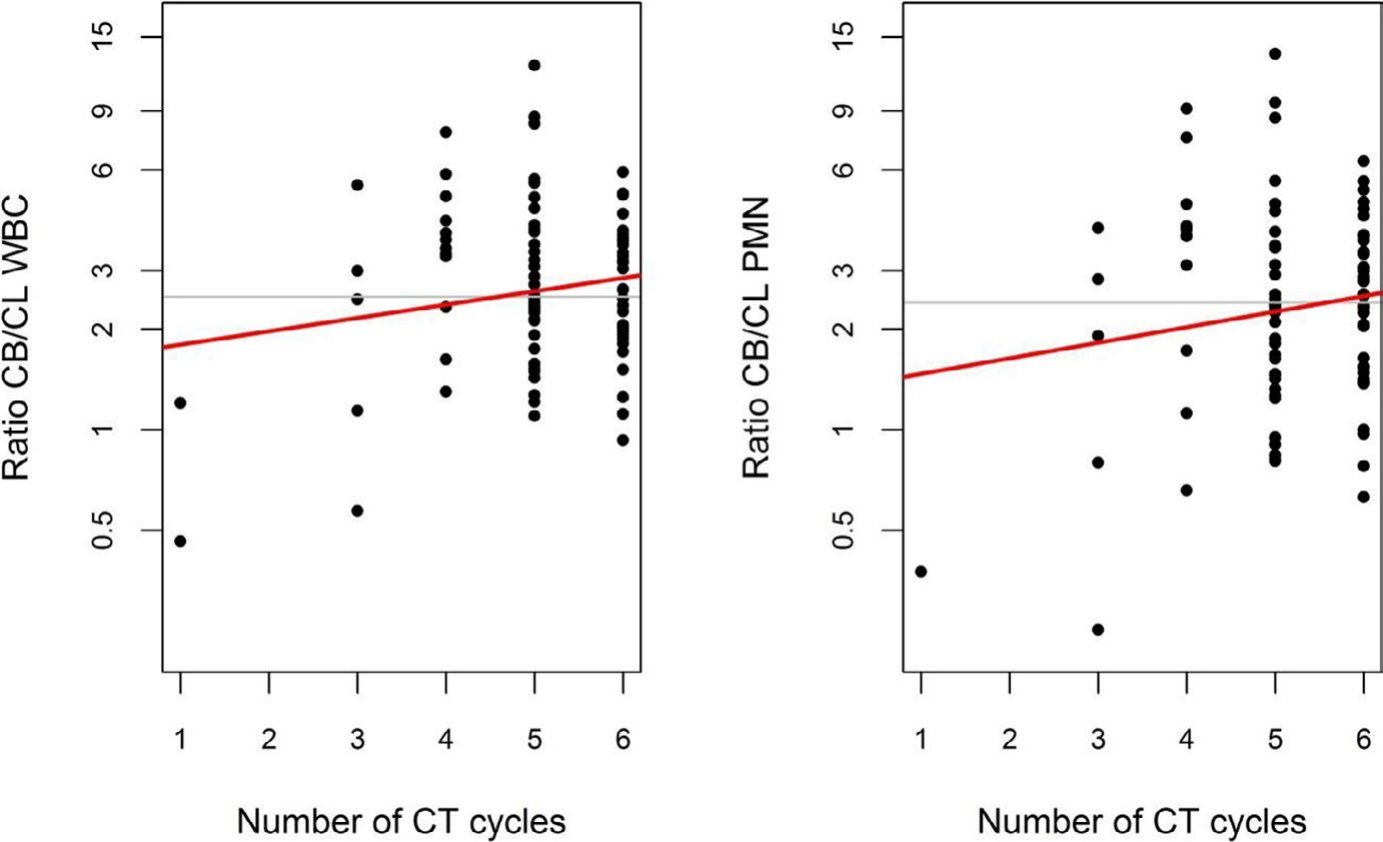
Relationship between the changes in the hematological parameters of WBC (left) and PMN (right) during CT, expressed as the ratio of hematological counts at baseline and the last CT cycle, and the actual number of CT cycles. The regression line is depicted in red, and the horizontal gray line is drawn at the median value of the ratio CB/CL (2.5 for WBC and 2.4 for PMN). CB baseline cycle; CL last cycle; WBC white blood cell; PMN polymorphonuclear neutrophil; CT chemotherapy

**Figure 2:**
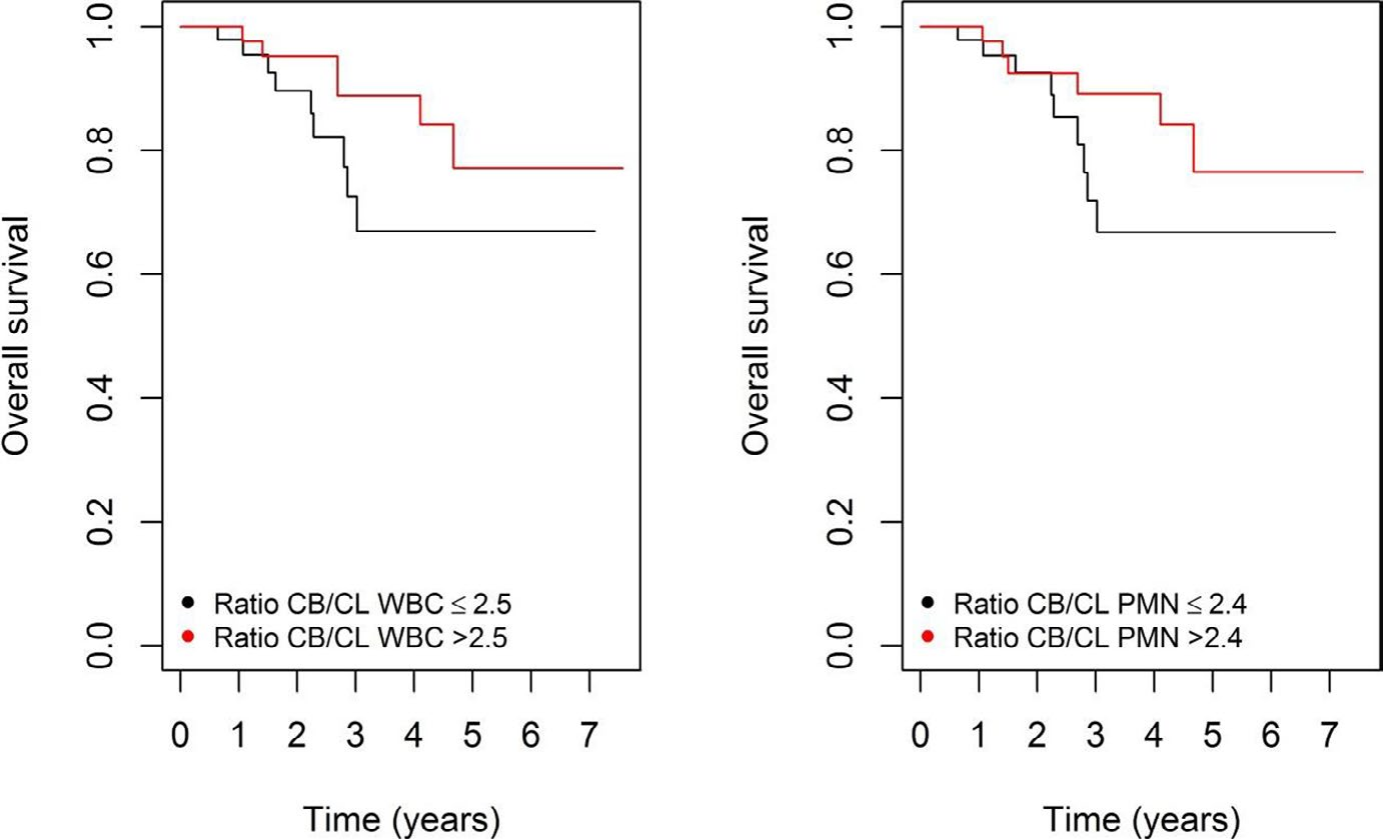
Kaplan‐Meier curves of overall survival according to the ratio CB/CL WBC above and below the median of 2.5 (left) and the ratio CB/CL PMN above and below the median of 2.4 (right). OS overall survival; CB baseline cycle; CL last cycle; WBC white blood cell; PMN polymorphonuclear neutrophil

In contrast, when considering the difference between the baseline and the last cycle of CT as defined in the statistical analysis section, a highly significant association was found between OS and WBCs (HR 0.29, 95% CI 0.11‐0.76, *P* = .011). The same observation applied to PMNs (HR 0.41, 95% CI 0.19‐0.89, *P* = .024) and lymphocytes (HR 0.35, 95% CI 0.15‐0.85, *P* = .020). For platelet count changes, only a tendency was noted (HR 0.34, 95% CI 0.11‐1.09, *P* = .069). For RFS, the findings were even more pronounced for WBCs (HR 0.35, 95% CI 0.17‐0.74, *P* = .0056) and PMNs (HR 0.44, 95% CI 0.24‐0.82, *P* = .0093) but not for lymphocytes (HR 0.71, 95% CI 0.33‐1.53, *P* = .38) or platelets (HR 0.43, 95% CI 0.17‐1.11, *P* = .083). A statistically significant association was also observed between FFDM and changes in WBCs (HR 0.37, 95% CI 0.16‐0.85, *P* = .020) and PMNs (HR 0.42, 95% CI 0.21‐0.84, *P* = .014). It is worth noting that the difference between the baseline and last CT cycles was moderately correlated with the actual number of CT cycles for both WBCs (r = 0.33, *P* = .0009) and PMNs (r = 0.32, *P* = .0015) (Figure 1). When combining the difference and the actual number of cycles in a Cox regression analysis of OS and RFS, the actual number of cycles never turned out to be significant, neither for WBCs nor for PMNs, reinforcing the predictive ability of the change in WBCs and PMNs from the first to the last CT cycle. As an illustration, Figure 2 displays the Kaplan‐Meier curves of OS for patients with a ratio CB/CL WBC above and below the median of 2.5 (left panel) and for patients with a ratio CB/CL PMN above and below the median of 2.4 (right panel).

## 4 DISCUSSION

Concomitant chemoradiation followed by IGBAT is the consensual treatment for patients presenting with locally advanced cervical neoplasia. The beneficial impact of such treatment has been demonstrated when compared to exclusive radiation. Studies dedicated to hematological changes during CCRT have mainly focused on the Hb profile. The potential link between WBC and PMN blood variations during treatment and patient outcomes remains to be clarified.

Our study population was homogeneous, with all women receiving cisplatin‐based CCRT followed by IGBAT. The observed 5‐year OS rate of 72% is comparable to that of the retroEMBRACE trial (OS rate of 66%).^23^ Smoking was found to be negatively related to OS, supporting findings of previous studies that demonstrated cigarette smoking decreases survival in LACC patients treated by RT or CCRT. ^24^, ^25^ Moreover, several epidemiological studies clearly established that smoking increases the risk of developing CC, by helping human papillomavirus survival and leading to the development of invasive lesions several years after initial infection.^26^, ^27^, ^28^ Tumor size is a well‐known negative prognostic factor in CC.^29^, ^30^ Here, it was found to be related to RFS even more than the FIGO 2009 classification. With a 1‐cm increase in tumor size, the risk of recurrence is multiplicated by a factor of 1.37 (relative increase of 37%).

This study also demonstrated that 26 patients (25.2%) at diagnosis and 85 patients (85.2%) during CCRT developed anemia according to the World Health Organization definition (Hb < 12 g/dL). Severe anemia (Hb < 8 g/dL) was rare (<2%). The median Hb count at diagnosis was negatively correlated with tumor size. In fact, this result and data from literature underlined that pretreatment but more frequently Hb count during RT was associated with other known prognostic factors such as tumor size, corpus invasion, nodal status, and FIGO stage.^31^, ^32^ No association was discerned between Hb count at diagnosis or during CCRT and OS, RFS, pelvic or distant metastases. In many tumors (eg, head and neck and cervical cancers), anemia has been associated with reduced local control and decreased survival. However, most retrospective studies concerned patients treated by RT alone, and data on tumor size were not included, thus inducing significant bias.^8^, ^9^, ^33^ Nevertheless, the associations between anemia and local recurrence and/or OS but also between hypoxia and radioresistance in animal models have led to the dogma that anemia increases tumor hypoxia in humans.^34^ Concerning cervical cancer, a positive impact of anemia correction by RBC transfusions was described by Bush^12^ in only one trial published in 1986. The authors concluded that maintaining a patient's Hb level above 12 g/dL throughout their treatment significantly improved pelvic control rates.^12^ The real effect of transfusion was not evaluable because of the heterogeneity and the small size of the population. Despite these weaknesses and one major retrospective trial that failed to confirm an association between anemia and recurrence, RBC transfusion still persists in daily practice.^35^ As in our trial, 85.2% of patients had Hb levels < 12 g/dL during CCRT, and applying the Bush^12^ principle to our patient population would have resulted in RBC transfusions in 85% of patients. In reality, 30 patients (31.3%) received RBC transfusions, and no association was found with outcomes (OS and RFS). Our study supports the conclusions of the trial published in 2015 by Bishop et al,^36^ who concluded, based on 678 patients treated by CCRT, that only a Hb level < 10 g/dL had a negative impact on disease‐specific survival. Here, such levels were rarely observed (4.8% at diagnosis and only 23.3% during CCRT) compared to those of Bishop et al^32^ (28% at diagnosis and 61% during CCRT). Bishop et al^36^ also demonstrated that 38% of patients received RBC transfusions without any impact on freedom from central recurrence and FFDM in the CCRT cohort but with a worsened outcome in patients treated with RT alone.

Animal models comfort our clinical findings demonstrating that a low Hb level did not influence intratumoral hypoxia and that reoxygenation occurring during treatment diminished the impact of hypoxia on the radiation response.^18^, ^37^ Kelleher et al^38^ explained that anemia correction with erythropoietin (EPO) or RBC transfusion partially reversed hypoxia in small tumors but not in larger ones. Sundfør et al^39^ described that only 50% of patients with CC increased their tumor oxygenation following transfusion. Furthermore, in a prospective trial on 20 patients treated by CCRT for head and neck SCC, RBC transfusions did not improve oxygenation and tumor outcomes. The authors concluded that RBC transfusions may be not useful.^40^ Concerning colorectal, bladder, and ovarian cancers, several large studies suggested an association between RBC transfusions in the perioperative period and an increased risk of cancer recurrence.^14^, ^15^, ^16^, ^41^, ^42^, ^43^, ^44^ Pro‐inflammatory and immunosuppressive effects (even in deleukocyte products) were brought up.^45^ Moreover, the risk of allergy and pathogen transmission although low still exists^46^, ^47^; some trials demonstrated a relationship with increased thrombotic risk.^48^ The cost of RBC transfusion must also be considered. In CC, the role of raising and maintaining Hb count by EPO was also addressed in two phase III randomized trials. First, Thomas et al^49^ evaluated this concept in patients treated by CCRT but this study was closed prematurely due to potential higher risk of thromboembolic event. Second, Blohmer et al^50^ investigated the effect of EPO in patients treated by surgery followed by CT and then RT. This study failed to demonstrate a significant benefit in survival outcomes. In addition, no significant differences in the safety profile were observed. Finally, a meta‐analysis in all cancer types showed an increase in mortality with erythropoiesis‐stimulating agents.^51^

Interestingly, our study highlights the positive impact of a WBC or PMN drop between the first and the last chemotherapy cycle during CCRT on outcomes (OS, RFS, and FFDM); the larger the drop in WBC or PMN counts, the better is the outcome. Moreover, a moderate association was found between these hematological variations and the number of CT cycles, indicating a potential association with a cumulative CT dose effect. When we combined the WBC or PMN drop from the first to the last cycle of CT and the actual number of CT cycles in a Cox regression model, the changes in WBCs or PMNs remained significantly related to outcome, in contrast to the actual number of CT cycles itself. This supports the concept of an intensity dose effect. Our results are in line with the recent publications of Wisdom et al^52^ and Yildirim et al^53^ on patients treated by CCRT and the current radiotherapeutic approach. Wisdom et al^52^ demonstrated that patients with higher neutrophil counts one week after the initiation of CCRT had reduced local and metastatic control. As in our study, this author did not report a correlation between the baseline PMN count and outcomes. In mouse models, it has also been reported that an experimental PMN count reduction before starting CCRT improves the radiation response and leads to the suppression of mitogen‐activated protein kinase transcriptional activity in tumor cells.^52^ Yildirim et al^53^ observed that the absolute neutrophil count after the 3rd week of CCRT was significantly higher in patients with progressive disease. Apart from these two trials, no other studies have investigated the association between PMN count and outcome during treatment in patients with LACC treated by CCRT, and no study but ours has explored a “dynamic” approach evaluating the relative change in PMN count during treatment.

In fact, the majority of the trials in CC, regardless of the setting, looked at the impact of pre‐treatment neutrophilia or the neutrophil‐to‐lymphocyte ratio on local relapses and survival. In most cases, these two variables were correlated with poorer outcomes.^54^, ^55^, ^56^, ^57^ In addition, two studies identified tumor‐associated neutrophils (TANs) as independent poor prognostic factor for survival, regardless of clinical stage or type of treatment.^58^, ^59^ Also, Matsumoto et al^59^ described that around 90% of CC with increased TANs were discovered in patients with pre‐treatment leukocytosis and neutrophilia.^59^ But, the precise relationship between circulating and tissue PMNs and the most pertinent factor remains unknown. In fact, a direct comparison does not exist. Concerning TANs, data on their function in human cancers are limited, mostly derived from animal models and only begun to be investigated recently. Moreover, there are no reliable standardized techniques for their assessment, leading to misleading results.^60^ More efficient methods are awaited. Furthermore, in our “WBC/PMN drop concept,” using TANs instead of PMN blood count, would require biopsy samples before and at the end of treatment. Thus, a blood PMN count seems currently the best choice, less expensive, and then blood PMN are easily separated from other immune cells by flow cytometry.^60^ Nevertheless, we can not affirm their similar functions. It is also important to highlight that our drop in WBC/PMN blood count could reflect an immunoresponse induced by CCRT. Indeed, RT exerts its anti‐tumoral effect through the induction of double‐strand DNA damage, leading to mitotic catastrophe, apoptosis, and senescence. More recently, preclinical and clinical data have suggested that RT can also produce immune‐stimulating effects through “immunogenic cell death (ICD).” This death of cancer cells induces the release of tumor‐associated antigens but also the production of “danger signals” also named damage‐associated molecular patterns such as calreticulin, high‐mobility group box 1protein (HMGB1), and adenosine triphosphate (ATP). They recruit and attract circulating myeloid cells (dendritic cells, macrophages, PMN,…) creating a pro‐inflammatory tumor TME.^19^ Many CT agents mediate their cytotoxic effects by the induction of apoptosis, initially thought to be non‐inflammatory and non‐immunogenic. In fact, CT drugs such as doxorubicin or oxaliplatin, are well described inducing this ICD. Conversely, the exact mechanism of immune response produced by cisplatin is less clear; for example, its incapacity for inducing calreticulin exposure was observed.^61^, ^62^, ^63^ Within the different immune cells present at the TME, myeloid cells are particularly important with their phagocytic ability, clearing dying cells. Thus, WBC/PMN is capted from blood toward tumor site. However, immune‐stimulating effects of RT are often masked by an immunosuppressive TME. Thus, an alternative approach to increase ICD is the concomitant use of RT and CT. This concept was confirmed by Golden et al^64^ in a mouse mammary adenocarcinoma cell line. In the future treatment of LACC, adding immune checkpoint inhibitors to RT will be potentially also a very attractive option. Tumor local control is probably the result of a combination of cytotoxic and immune effects of CT and RT. In another mammary cell model, hosts providing a major immune response at the primary tumor site could also stimulate a systemic immune reaction targeting distant micrometastases, with impact on survival outcomes.^65^, ^66^

An abundance of literature reports the positive impact of chemotherapy‐induced neutropenia (CIN) on outcome in several types of cancers treated by CT in a metastatic or an adjuvant setting, underlining the important implication of PMN in cancer biology.^21^ Indeed, Shitara et al^67^ demonstrated, in a meta‐analysis of 9528 patients, that high‐grade neutropenia or leukopenia occurring during CT in patients with several types of malignancies was associated with a 31% reduction in the risk of death. However, as far as cervical cancer is concerned, there is a clear lack of data about CIN in advanced disease treated by CCRT followed by IGBAT. Only one publication confirmed the positive impact of CIN in patients treated by surgery followed by CCRT.^68^ The concept of CIN is in agreement with our results about the importance of PMNs on chemo‐ and/or radiotherapy responses, especially their positive impact when PMN levels are low. Nevertheless, there is no consensus regarding the optimal way to implement PMN variables in clinical practice. Whether the absolute PMN count or change in PMN blood counts during CT cycles should be used is unclear and should be addressed in prospective clinical trials. ﻿Neutropenia is too often seen only as a predictor of infectious complications after CT because neutrophils are some of the earliest cells responding to sites of tissue injury and infection. Nevertheless, they can have pro‐ and anti‐ tumoral effects in many ways (angiogenesis, immunity, and tumor cell proliferation) controlled by signals from cancer cells or their microenvironment.^69^ Other hypothetical underlying mechanisms of the impact of CIN and our results are based on cancer stem cells and underdosing theories. The positive effect of CIN could reflect the response of hematopoietic stem cells to CT, which partly depends on the drug concentration. Because some subpopulations of tumor cells have gained normal stem cell properties, including self‐renewal, differentiation, metastasis dissemination, and anti‐apoptosis, it has been suggested that with increasing severity of neutropenia, a greater proportion of cancer stem cells will be killed, hence potentially improving survival. Further work is required to determine whether the severity (rather than just the occurrence) of neutropenia correlates with clinical outcome.^70^ The last theory is concerned with the fact that the absence of neutropenia or PMN drop could mean an underdosing of chemotherapy. It is well known that there are multiple negative conclusions about the correlation between body surface area and pharmacokinetic variables because of significant interpatient variability in metabolism (variability in drug concentration within the tumor, the microenvironment of tumor cells and genetic variation‐polymorphism in both the patient (germline) and the tumor). Therefore, prescribing CT using body surface area lacks the precision to be sure that all patients receive an adequate CT dose.^71^, ^72^ Other teams evaluated the tailoring of the CT dose according to neutrophil counts, notably in breast but also gastric cancers.^73^, ^74^ All these findings suggest that the explanation for a positive association between patient outcome and a substantial drop in WBC or PMN blood counts during CT cycles is a very complex issue. In LACC, there is also a need for new biomarkers to better determine which patients would be at particular risk of distant failure requiring stricter follow‐up and perhaps intensification of systemic treatments. The change in PMN between the first and last cycles of CT may be an interesting new biomarker since it correlates with RFS, OS, and distant recurrence. It could potentially be combined with other well‐known pre‐treatment predictive markers (tumor size, node disease) to envisage adjuvant systemic treatments (CT or new drugs as immunotherapy) if the ongoing trials confirm their positive impact prospectively. It could also be considered to intensify follow‐up as is performed with patients who have positive lymph nodes.

We acknowledge the retrospective nature of the study, which entails potential biases. Missing data could not be retrieved, and outlying observations could not be double‐checked. However, all patients included fulfilled a number of strict inclusion and exclusion criteria to constitute a homogeneous group of patients with LACC. All patients were treated in the same institutional network, but a prospective multi‐institutional approach would be needed to confirm the robustness of our prognostic variables in a prospective study.

In conclusion, we showed that a major drop in WBC and PMN counts between the first and last cycles of CT treatment positively impacted OS, RFS, and the rate of distant metastasis. To the best of our knowledge, this is the first study depicting PMN blood count changes during CCRT as a predictive factor for outcome in LACC. However, there is a need for further research in a prospective setting to confirm the usefulness of the new inexpensive biomarker. The latter might be used to adapt our follow‐up intensity and to consider systemic adjuvant treatments in case of other “borderline” risk factors. Furthermore, our study found no association between the Hb count, either at diagnosis or during treatment, or RBC transfusions and outcome. These findings shed light on the controversial management of anemia in patients with LACC during concomitant chemoradiation. The common practice is to transfuse RBCs to maintain Hb around 12 g/dL, but a stricter attitude aimed at maintaining Hb around 10 g/dL may be recommended, balanced by other potential risks of RBC transfusion.

## CONFLICT OF INTEREST

The authors declare no conflicts of interest.

## AUTHORS’ CONTRIBUTIONS

Conception/Design: Christine Gennigens, Guy Jerusalem, Frédéric Kridelka. Provision of study materials or patients: Christine Gennigens, Marjoleine De Cuypere Annelore Barbeaux, Frédéric Forget, Johanne Hermesse, Frédéric Kridelka. Collection and assembly of data: Christine Gennigens. Data analysis and interpretation: Christine Gennigens, Guy Jerusalem, Frédéric Kridelka Adelin Albert, Laurence Seidel. Manuscript writing: Christine Gennigens, Guy Jerusalem, Adelin Albert, Frédéric Kridelka.

## ETHICS APPROVAL AND CONSENT TO PARTICIPATE

The Institutional Review Board of the University Hospital of Liège (Belgium) approved the work, and the requirement for written informed consent was waived.

## CONSENT FOR PUBLICATION

Not applicable. This report does not contain any individual person's information.

## Data Availability

All the data are included in an Excel file.
